# Structural insights into the binding mode of the hypnotic drug vornorexant to orexin receptors

**DOI:** 10.1107/S2053230X26003432

**Published:** 2026-04-30

**Authors:** Masashi Mima, Chiemi Mishima-Tsumagari, Mayumi Sakata-Sakuta, Ryo Iwaoka, Kunihiko Iwamoto

**Affiliations:** a Taisho Pharmaceutical Co. Ltd 1-403 Yoshino-cho, Kita-ku Saitama331-9530 Japan; University of Toronto, Canada

**Keywords:** vornorexant, orexin receptors, crystal structure, dual orexin receptor antagonists, U-shaped conformation

## Abstract

Insomnia is a widespread sleep disorder that significantly impairs quality of life and imposes a societal burden. Although benzodiazepines and Z-drugs are commonly used for treating insomnia, these drugs often have side effects, such as excessive muscle relaxation and dependency. The orexin signaling pathway has emerged as a promising therapeutic target for insomnia, with dual orexin receptor antagonists (DORAs) offering an alternative approach to treatment. To reduce the potential of these drugs for next-day residual effects, we developed vornorexant, a novel DORA with a high receptor affinity and a short elimination half-life. In this study, we investigated the molecular interactions of this drug with human orexin receptors through crystal structure analysis of its binding to orexin type 2 receptor (OX_2_R) and a docking simulation of the drug with orexin type 1 receptor (OX_1_R). The crystal structure of the OX_2_R–vornorexant complex revealed a conserved U-shaped conformation stabilized by hydrophobic and hydrogen-bonding interactions, including key contacts with Asn324, His350 and Pro131. OX_1_R–vornorexant docking simulations indicated a similar binding mode to OX_1_R, with no steric hindrance observed, supporting a balanced dual antagonism. These results provide a structural basis for the high-affinity dual antagonism of vornorexant and offer insights for the design of orexin receptor antagonists.

## Abstract 2

The crystal structure of the human orexin type 2 receptor–vornorexant complex (3.29 Å resolution) and orexin type 1 receptor docking revealed a conserved U-shaped binding conformation, explaining the high affinity and balanced dual antagonism of this dual orexin receptor antagonist. The structural data validate the rational design that incorporated a 1,3-oxazinan ring and pyrazole moiety to reduce lipophilicity while maintaining strong receptor affinity, highlighting the potential of vornorexant as an effective insomnia treatment with a lower risk of next-day residual effects.

## 1. Introduction

Insomnia is one of the most prevalent sleep disorders, significantly impairing individuals’ quality of life and imposing a substantial burden on society (Ozminkowski *et al.*, 2007 ▸; Kessler *et al.*, 2011 ▸). Conventional treatments have relied on benzodiazepines and Z-drugs that positively modulate the activity of the gamma-aminobutyric acid (GABA)_A_ receptor, but despite their efficacy these drugs can exert adverse effects such as excessive muscle relaxation and dependency (Baldwin *et al.*, 2013 ▸; Umbricht & Velez, 2021 ▸).

In recent years, the orexin–orexin receptor signaling pathway has emerged as a promising therapeutic target for insomnia. Orexin, a neuropeptide produced in the lateral hypothalamus, exerts its effects through binding to two receptors, the orexin type 1 receptor (OX_1_R) and orexin type 2 receptor (OX_2_R), which are widely distributed throughout the central nervous system and play a crucial role in maintaining wakefulness (de Lecea *et al.*, 1998 ▸; Chemelli *et al.*, 1999 ▸; Lin *et al.*, 1999 ▸; Thannickal *et al.*, 2000 ▸; Messina *et al.*, 2017 ▸; Sakurai *et al.*, 1998 ▸). Orexinergic neurons project to monoaminergic and cholinergic nuclei in the brainstem and hypothalamus, promoting arousal by activating neurotransmitters such as histamine, dopamine and serotonin (Sakurai, 2007 ▸). Between the two types of orexin receptors, OX_2_R is considered central to the regulation of wakefulness, as OX_2_R-deficient mice have been shown to exhibit narcolepsy-like symptoms (Chemelli *et al.*, 1999 ▸). OX_1_R plays a supplementary role, and the absence of both receptors leads to more severe phenotypes (Sakurai, 2007 ▸; Willie *et al.*, 2003 ▸; Hasegawa *et al.*, 2014 ▸).

Hyperactivation of orexin signaling has been implicated in the pathophysiology of insomnia (Chow *et al.*, 2016 ▸; Mieda & Sakurai, 2013 ▸), and dual orexin receptor antagonists (DORAs) have gained attention as novel therapeutic agents. Currently, DORAs such as suvorexant (Cox *et al.*, 2010 ▸), lemborexant (Yoshida *et al.*, 2015 ▸) and daridorexant (Treiber *et al.*, 2017 ▸) are approved for clinical use. However, these DORAs have a relatively long elimination half-life, which may raise concerns about next-day residual effects in certain groups of patients, such as elderly patients. Suvorexant at high doses (*e.g.* 40 mg per day) has been reported to be associated with significant carryover effects, leading the US Food and Drug Administration (FDA) to limit approval to a maximum dose of 20 mg per day (Citrome, 2014 ▸).

To address this limitation of a long elimination half-life, we developed a novel DORA, vornorexant, which combines high receptor affinity with the shortest elimination half-life of the DORAs currently available on the market (Hikichi *et al.*, 2025 ▸; Konno *et al.*, 2024 ▸). In general, shortening the elimination half-life requires a reduction in the volume of distribution, which is typically achieved by decreasing the lipophilicity of the compound. However, since orexin receptor binding is predominantly driven by hydrophobic interactions (Suno *et al.*, 2018 ▸), reducing the lipophilicity of a drug could be expected to compromise its binding affinity. We overcame this challenge in vornorexant by incorporating a 1,3-oxazinan ring and a pyrazole moiety to achieve a balance between low lipophilicity and high receptor affinity (Suzuki *et al.*, 2015 ▸; Futamura *et al.*, 2020 ▸). In a phase I clinical trial conducted in healthy subjects, vornorexant exhibited rapid absorption and a short half-life (Kambe *et al.*, 2023 ▸). In a phase IIa trial, significant improvements in both objective and subjective parameters of sleep were reported in patients with insomnia (Uchiyama *et al.*, 2022 ▸).

In this study, we attempted to elucidate how the structural features contributing to the low lipophilicity of vornorexant interact with OX_1_R and OX_2_R. To this end, we conducted complex crystal structure analysis of the binding of the drug to human OX_2_R and performed complex structure modeling through docking simulations of the drug with human OX_1_R.

## 2. Materials and methods

### 2.1. Protein production and purification

The construct of human OX_2_R for crystallization used the sequence of PDB entry 7xrr as a reference (Asada *et al.*, 2022 ▸). Plasmids encoding human OX_2_R with a hemagglutinin (HA) signal peptide and FLAG tag followed by an HRV 3C protease cleavage site attached at the N-terminus, and an HRV 3C protease cleavage site followed by a His_10_ tag at the C-terminus, in the mammalian expression vector pcDNA3.4 were obtained from GenScript. The codon usage was optimized for expression in mammalian cells. The expression plasmids were transfected into Expi293F cells (Thermo Fisher Scientific) using ExpiFectamine 293 (Thermo Fisher Scientific), in accordance with the manufacturer’s instructions, and the transfected cells were cultured in Expi293 Expression Medium (Thermo Fisher Scientific) for three days in a shaking flask. The cells were harvested and resuspended in hypotonic buffer (10 m*M* HEPES pH 7.5, 10 m*M* NaCl, 10 m*M* MgCl_2_), followed by homogenization and centrifugation (100 000*g*, 30 min, 4°C). The pellets were collected and washed twice to prepare membranes using a high-osmotic buffer (10 m*M* HEPES pH 7.5, 500 m*M* NaCl, 10 m*M* MgCl_2_). The washed membrane was solubilized by incubation (2 h, 4°C) with solubilization buffer [20 m*M* HEPES pH 7.5, 750 m*M* NaCl, 5 m*M* imidazole, 1%(*w*/*v*) *n*-dodecyl-β-d-maltopyranoside (DDM; Anatrace), 0.2% sodium cholate (FUJIFILM Wako), 0.2% cholesteryl hemisuccinate Tris salt (CHS; Anatrace), 2 mg ml^−1^ iodoacetamide (Sigma–Aldrich), 1× protease inhibitor cocktail (Roche), 100 µ*M* vornorexant (synthesized at Taisho)]. The supernatant was isolated by ultracentrifugation for 30 min at 150 000*g* and captured using an Ni–NTA agarose column (Qiagen). The column was sequentially washed with three buffers, wash buffer 1 [20 m*M* HEPES pH 7.5, 750 m*M* NaCl, 20 m*M* imidazole, 0.1%(*w*/*v*) DDM, 0.02% sodium cholate, 0.02% CHS, 50 µ*M* vornorexant], followed by wash buffer 2 [20 m*M* HEPES pH 7.5, 750 m*M* NaCl, 20 m*M* imidazole, 0.1%(*w*/*v*) lauryl maltose neopentyl glycol (LMNG; Anatrace), 0.02% sodium cholate, 0.02% CHS, 50 µ*M* vornorexant] and finally wash buffer 3 [20 m*M* HEPES pH 7.5, 750 m*M* NaCl, 40 m*M* imidazole, 0.01%(*w*/*v*) LMNG, 0.002% sodium cholate, 0.002% CHS, 50 µ*M* vornorexant], and the bound protein was eluted with elution buffer [20 m*M* HEPES pH 7.5, 750 m*M* NaCl, 500 m*M* imidazole, 0.01%(*w*/*v*) LMNG, 0.002% sodium cholate, 0.002% CHS, 50 µ*M* vornorexant]. HRV 3C protease (home-made) was added to the eluate, followed by incubation overnight at 4°C. HRV 3C protease was then removed using Glutathione-Sepharose FF. The protein was concentrated in a 50 kDa cutoff Amicon Ultra concentrator (Millipore) and run on an Superdex 200 Increase size-exclusion column (Cytiva) in SEC buffer [20 m*M* HEPES pH 7.5, 100 m*M* NaCl, 0.01%(*w*/*v*) LMNG, 0.002% sodium cholate, 0.002% CHS, 50 µ*M* vornorexant]. The peak fractions were collected and 0.2 m*M* vornorexant was added. The protein was concentrated to approximately 50 mg ml^−1^ with a 50 kDa cutoff Amicon Ultra concentrator.

### 2.2. Crystallization, data collection and structure determination

Purified OX_2_R was mixed with 1.5 parts by weight of monoolein (Nu-Check) containing 10%(*w*/*w*) cholesterol (Sigma) using the syringe-reconstitution method (Caffrey & Cherezov, 2009 ▸). The lipidic cubic phase mix was dispensed in 100 nl drops onto glass plates and overlaid with 1000 nl precipitant solution using NT8 (Formulatrix). Crystallization experiments were carried out in 96-well glass sandwich plates (Hampton Research), which were incubated at 20°C. Crystals of the OX2R–vornorexant complex were grown in precipitant solution A6 of JBScreen LCP HTS (Jena Bioscience GmbH) consisting of 150 m*M* ammonium sulfate, 100 m*M* HEPES pH 7.5, 10%(*v*/*v*) PEG 400 and the crystals were flash-cooled at a temperature of 100 K. Diffraction data were collected using multiple microcrystals at SPring-8 (Hyogo, Japan) and data processing was performed using *XDS* (Kabsch, 2010 ▸) from the *CCP*4 suite (Agirre *et al.*, 2023 ▸). The crystal structures were determined by molecular replacement with *MOLREP* (Vagin & Teplyakov, 1997 ▸) using a published structure (PDB entry 7xrr) as the search model. The structure was refined with *REFMAC*5 (Murshudov *et al.*, 1997 ▸). Interactive graphical model building was carried out with *Coot* (Emsley & Cowtan, 2004 ▸). The data-collection and refinement statistics are summarized in Table 1 ▸.

### 2.3. Modeling

Docking simulations of the OX_1_R–vornorexant complex were performed using the *Molecular Operating Environment* (*MOE*) software platform (Molecular Operating Environment, Chemical Computing Group, Montreal, Canada), employing the Amber19:EHT force field and generalized Born approximation. Initially, the crystal structure of the OX_2_R–vornorexant complex was prepared (structural issue correction, protonation and partial charge assignment) for homology modeling and analysis. A stable conformation of vornorexant retrieved from the crystal structure was searched for using the *LowModeMD* method (MOE/Conformation/Search) and subsequently the lowest energy conformation was selected as the input for the docking simulation. The 3D structure of OX_1_R was modeled based on the crystal structure of OX_2_R (MOE/svl:pro_Model) without its missing regions. The model structure of OX_1_R underwent energy minimization, focusing solely on the side chains. Finally, *SiteFinder* was executed to define the orthosteric site of OX_1_R, and the docking simulation was performed by generating a thousand poses and outputting a hundred refined poses. The pose with the lowest ligand score was selected for further analysis.

## 3. Results and discussion

### 3.1. Overall structure and binding mode of vornorexant to OX_2_R

**Figure 1:**
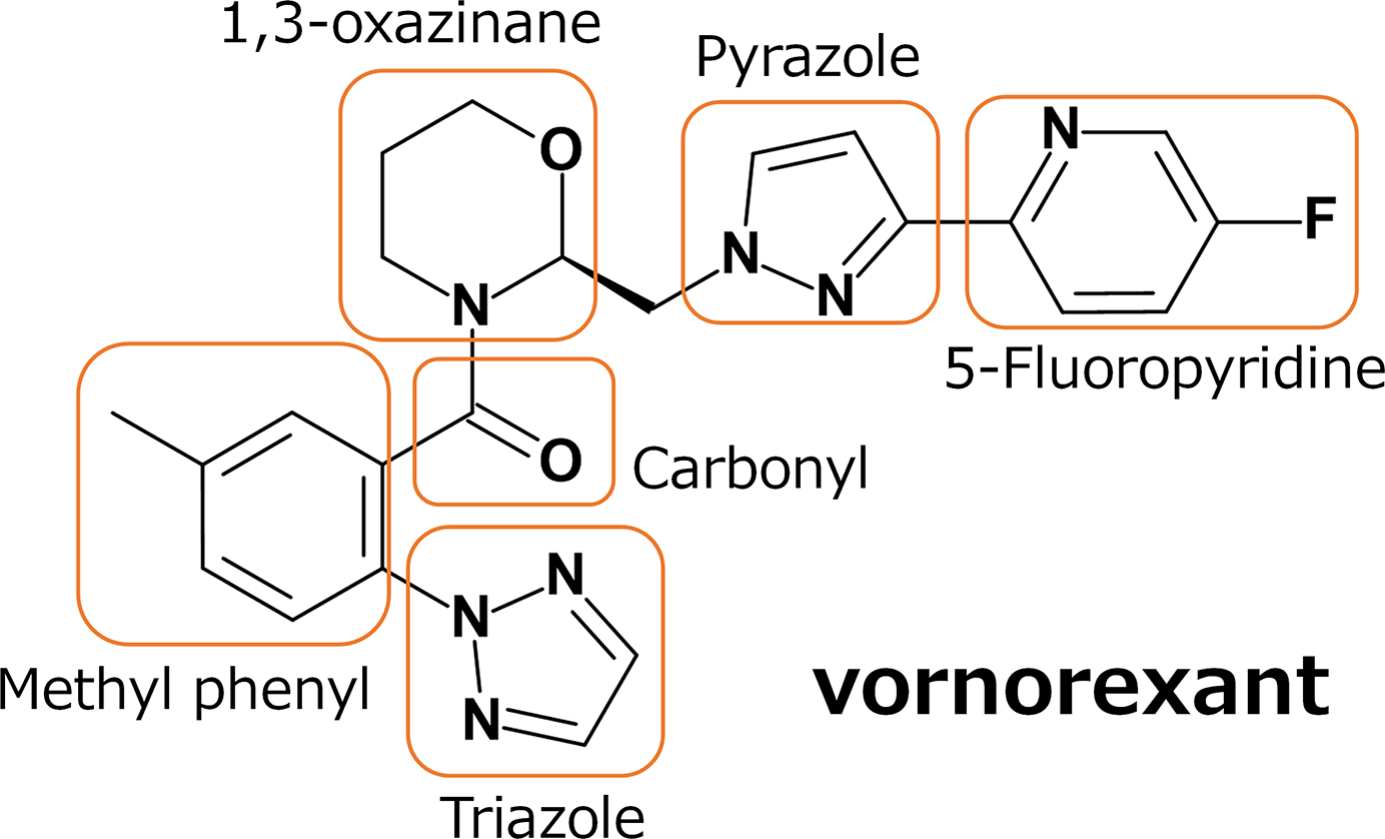
Chemical structure of vornorexant.

**Figure 2:**
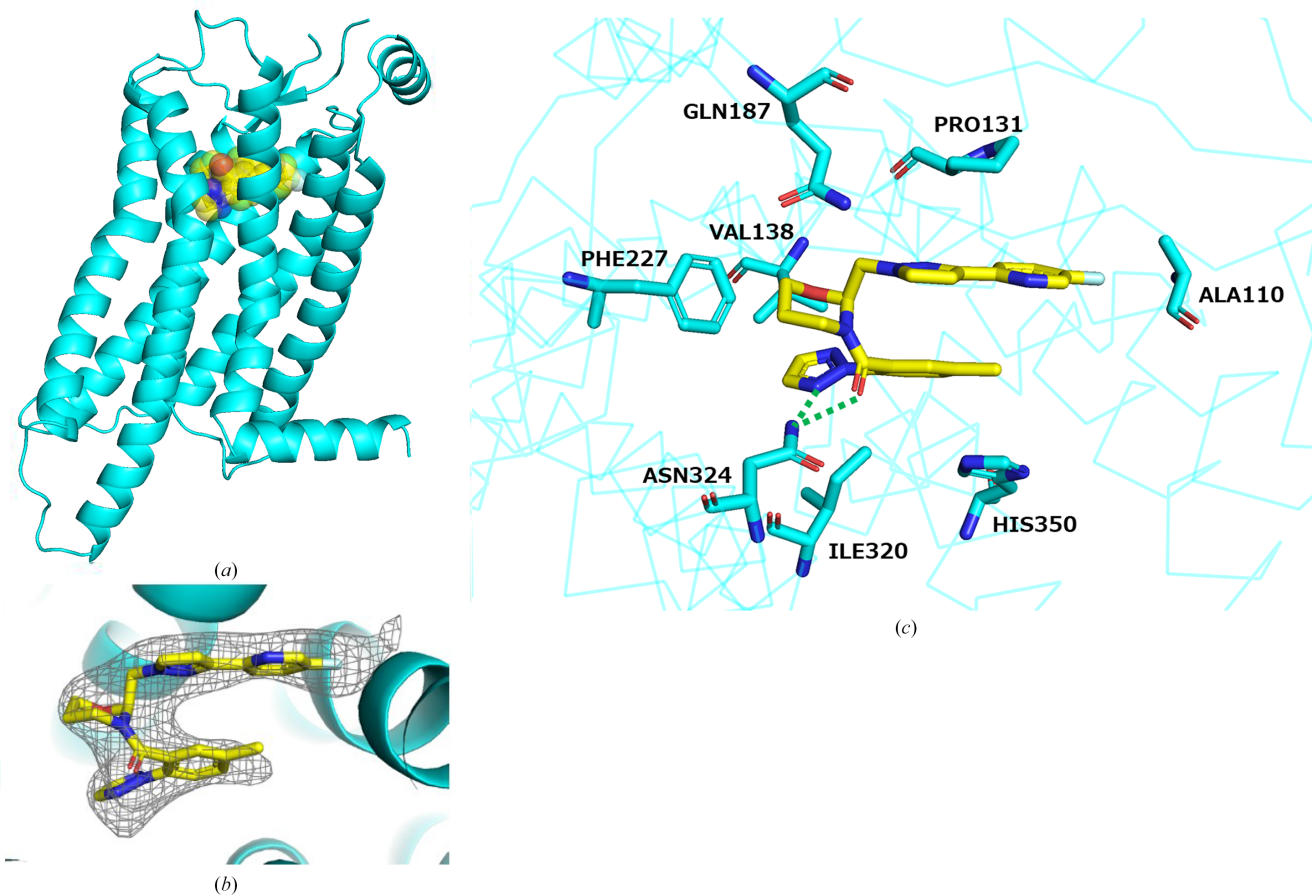
Crystal structure of the vornorexant–OX_2_R complex. (*a*) Overall structure of the vornorexant–OX_2_R complex. The OX_2_R molecule is shown in blue and vornorexant is shown in yellow using a Corey–Pauling–Koltun (CPK) space-filling model. (*b*) Electron density of vornorexant in the binding pocket of OX_2_R. The ligand-omit electron density is displayed as a gray mesh for the *F*_o_ − *F*_c_ map contoured at 3σ. (*c*) Binding mode of vornorexant with OX_2_R. Vornorexant and its interacting residues are represented as stick models, with C atoms shown in yellow (vornorexant) and blue (OX_2_R). Hydrogen bonds are indicated by green dotted lines.

Vornorexant (Fig. 1 ▸), an orexin receptor antagonist, has emerged as a promising therapeutic candidate for insomnia due to its high affinity for the orexin receptors and its short elimination half-life of 1.32–3.25 h (Kambe *et al.*, 2023 ▸). These properties are expected to reduce next-day residual effects while allowing the sleep-promoting efficacy to be maintained throughout the major part of the night. In this study, we conducted a crystallographic analysis of human OX_2_R complexed with vornorexant to elucidate the molecular binding mode. The crystal structure of the OX_2_R–vornorexant complex was determined at a resolution of 3.29 Å, which revealed detailed interactions between the ligand and the receptor. The arrangement of the transmembrane helices in OX_2_R closely resembled those in previously reported antagonist-bound structures with suvorexant and lemborexant (Asada *et al.*, 2022 ▸; Yin *et al.*, 2015 ▸), indicating that OX_2_R adopts a pharmacologically relevant antagonist-bound conformation (Figs. 2 ▸*a* and 2 ▸*b*). Vornorexant bound to the orthosteric binding site of OX_2_R, which was consistent with the reported pharmacological data that vornorexant inhibits radioligand binding to the orexin receptors in a competitive manner (Hikichi *et al.*, 2025 ▸). Within the orthosteric binding site of OX_2_R, vornorexant adopted a U-shaped conformation, stabilized by the chair-like geometry of its 1,3-oxazinan ring. The axial orientation of the substituent at position 2 and the equatorial orientation at position 3 facilitated π–π stacking interactions between the methylphenyl and fluoropyridyl-pyrazole moieties (Fig. 2 ▸*c*). These interactions contribute to the stabilization of the three-dimensional structure of the ligand and enhance its binding affinity to OX_2_R. In this U-shaped conformation, vornorexant is anchored by a hydrogen bond to Asn324 and hydrophobic packing that flanks the ligand between Pro131 and His350 and contacts Ile320 (Fig. 2 ▸*c*). The 1,3-oxazinan ring makes van der Waals contacts with Phe227, and the triazole moiety makes close van der Waals contacts with Val138 and Ile320 (Fig. 2 ▸*c*). Together, these contacts indicate that vornorexant is anchored by conserved hydrophobic packing around the methylphenyl-triazole region (flanked by Pro131/His350 and contacting Ile320), while the lipophilicity-reducing heterocycles contribute additional local stabilization through close packing and polar interactions. Thus, despite the introduction of lipophilicity-reducing heterocycles, vornorexant preserves a pocket-spanning hydrophobic packing mode that is also observed for other DORAs in OX_2_R, and local polarity- and geometry-driven interactions, including hydrogen bonding to Asn324 and shape complementarity of the chair-like 1,3-oxazinan ring, help maintain high affinity without requiring expansion of the overall hydrophobic surface area of the ligand.

### 3.2. Comparison of the binding modes of vornorexant to OX_1_R and OX_2_R

**Figure 3:**
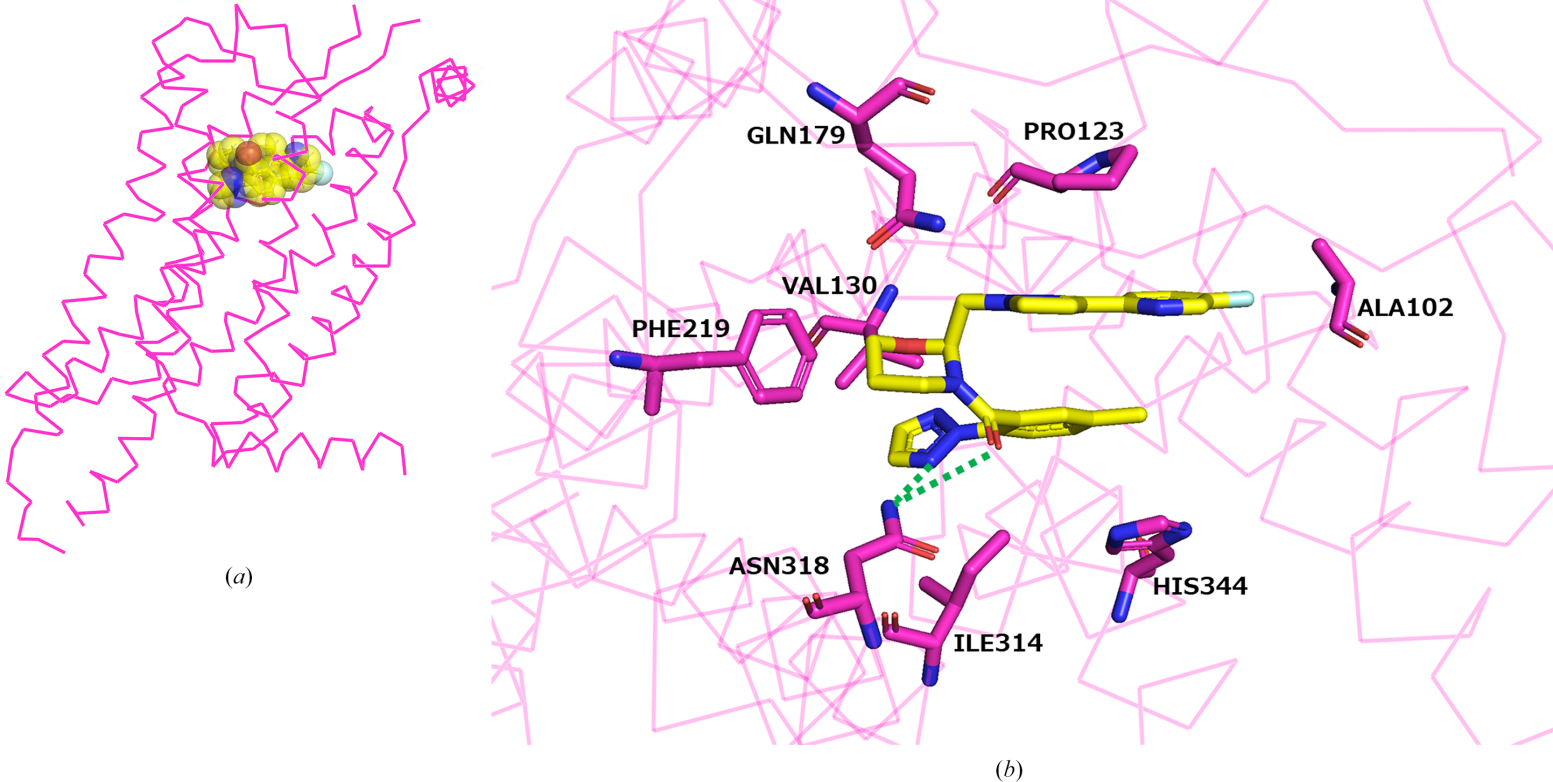
Docking simulation model of the vornorexant–OX_1_R complex. (*a*) Overall structure of the vornorexant–OX_1_R complex. The OX1R molecule is shown in magenta and vornorexant is shown in yellow using a CPK space-filling model. (*b*) Binding mode of vornorexant within OX_1_R. Vornorexant and the interacting residues of OX_1_R are represented as stick models, with C atoms in yellow (vornorexant) and magenta (OX_1_R) color. Hydrogen bonds are indicated by green dotted lines.

Vornorexant is a DORA that exhibits inhibitory activity against both OX_1_R and OX_2_R. To better understand its pharmacological profile, it is essential to elucidate its binding modes with both receptor subtypes. Based on the crystal structure of the OX_2_R–vornorexant complex, we performed a docking simulation to construct a modeled complex structure of vornorexant bound to human OX_1_R (Fig. 3 ▸*a*). Comparison of the modeled OX_1_R–vornorexant complex with the experimentally determined OX_2_R structure revealed that vornorexant adopts a similar U-shaped conformation upon binding to OX_1_R, consistent with its conformation in the OX_2_R complex (Fig. 3 ▸*b*). The key interacting residues in both receptors were largely conserved, and the overall interaction patterns exhibited a high degree of similarity (Figs. 2 ▸*c* and 3 ▸*b*). Unlike the case reported for lemborexant (Asada *et al.*, 2022 ▸), where steric hindrance involving Thr135 in OX_2_R (corresponding to Ala127 in OX_1_R) leads to differences in the binding modes of the drug between the two receptors, no such steric effects were observed with vornorexant. This absence of steric interference supports the structural compatibility of vornorexant with both receptor subtypes. These findings are consistent with the reported pharmacological data, indicating that vornorexant exhibits nearly equivalent inhibitory activity against both OX_1_R and OX_2_R (Hikichi *et al.*, 2025 ▸). The conserved binding conformation and interaction profile across both receptors further validate the molecular design strategy adopted with the aim of achieving balanced dual antagonism while minimizing the lipophilicity.

### 3.3. Comparison of the binding modes of vornorexant, suvorexant and lemborexant

**Figure 4:**
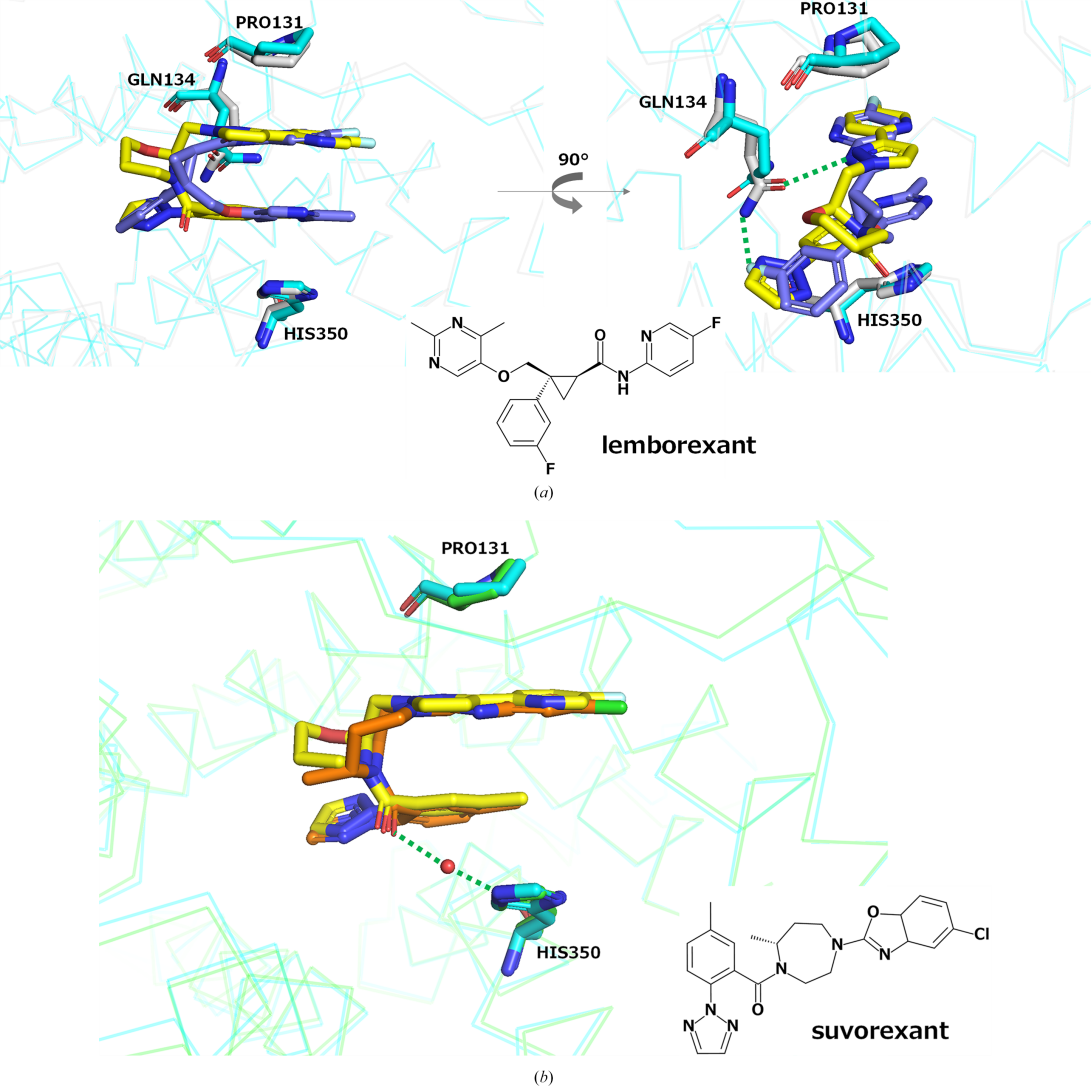
Comparison of the binding modes of antagonists to the orexin receptors. Superimposed crystal structures showing the binding modes of vornorexant (yellow) to OX_2_R (blue), compared with (*a*) lemborexant (deep blue) in the lemborexant–OX_2_R complex (PDB entry 7xrr) and (*b*) suvorexant (orange) in the suvorexant–OX_2_R complex (PDB entry 4s0v). Hydrogen bonds are indicated by green dotted lines.

To further evaluate the binding characteristics of vornorexant to OX_2_R, we conducted a structural superposition analysis of the previously reported crystal structures of the OX_2_R–suvorexant complex (PDB entry 4s0v; Yin *et al.*, 2015 ▸) and the OX_2_R–lemborexant complex (PDB entry 7xrr; Asada *et al.*, 2022 ▸) with the newly determined OX_2_R–vornorexant complex. Vornorexant was found to occupy the same ortho­steric binding site within OX_2_R as suvorexant and lemborexant, adopting a U-shaped conformation nestled between the side chains of Pro131 and His350 in all three complexes (Figs. 4 ▸*a* and 4 ▸*b*). This conserved structural feature is considered to be the key determinant of the high binding affinity observed across these three DORAs. The most notable difference in comparison with lemborexant was the involvement of the Gln134 residue (Fig. 4 ▸*a*). While lemborexant forms two hydrogen bonds to the side chain of Gln134, vornorexant did not engage with this residue as the side chain of Gln134 is oriented away from the ligand in the vornorexant-bound structure (Fig. 4 ▸*a*). In the comparison with suvorexant, the positioning of the carbonyl group adjacent to the common methylphenyl-triazole moiety was nearly identical in both compounds (Fig. 4 ▸*b*). However, suvorexant forms a water-mediated hydrogen bond between the carbonyl O atom and His350, whereas no such interaction was observed in the vornorexant complex (Fig. 4 ▸*b*). This absence may be attributable to the inadequate resolution of the crystal structure, and future high-resolution structural analyses may reveal the presence of such water molecules.

## 4. Conclusion

In this study, we elucidated the binding modes of vornorexant to OX_1_R and OX_2_R and showed that it adopts a conserved U-shaped conformation in the orthosteric pockets of both receptors. Conserved hydrophobic packing, together with key hydrogen-bonding interactions, stabilizes this pose and provides a structural basis for the high affinity and balanced antagonism of vornorexant across receptor subtypes. Across both receptors, lipophilicity-reducing heterocycles can be accommodated without disrupting the conserved interaction pattern required for tight binding. Collectively, these findings clarify the molecular basis of vornorexant binding and facilitate the structure-based interpretation of its pharmacological profile. 

## Supplementary Material

PDB reference: human orexin type 2 receptor in complex with vornorexant, 21ci

## Figures and Tables

**Table 1:** X-ray data-collection and refinement statistics for the vornorexant–OX_2_R complex (PDB entry 21ci) Values in parentheses are for the highest resolution shell.

Data collection	
Diffraction source	BL32XU, SPring-8
Space group	*P*2_1_2_1_2_1_
*a*, *b*, *c* (Å)	49.46, 90.88, 111.54
α, β, γ (°)	90, 90, 90
Wavelength (Å)	1.0000
Resolution limits (Å)	70.46–3.29 (3.49–3.29)
*R*_meas_ (%)	133.8
Total No. of reflections	614547 (80188)
No. of unique reflections	8106 (1276)
〈*I*/σ(*I*)〉†	10.91 (0.58)
CC_1/2_	0.990 (0.552)
Completeness (%)	99.9 (100.0)
Multiplicity	75.8 (62.8)
Wilson *B* factor (Å^2^)	63.64
Refinement	
Resolution (Å)	47.58–3.29
*R*_work_/*R*_free_ (%)	24.3/29.5
No. of non-H atoms	
Protein	2520
Ligand	33
Sulfate	10
Average *B* factors (Å^2^)	
Protein	67.910
Ligand	54.673
Sulfate	82.530
R.m.s.d.	
Bond lengths (Å)	0.005
Angles (°)	1.471
Ramachandran plot	
Most favored (%)	98
Allowed (%)	2
Outliers (%)	0

† CC_1/2_ was used as the cutoff for the resolution limit, and the mean *I*/σ(*I*) falls below 2.0 at 3.57 Å.

## Data Availability

The crystal structure of OX_2_R–vornorexant has been deposited in the Protein Data Bank as PDB entry 21ci.
